# Reactive Oxygen Species and Nitric Oxide Control Early Steps of the Legume – *Rhizobium* Symbiotic Interaction

**DOI:** 10.3389/fpls.2016.00454

**Published:** 2016-04-08

**Authors:** Isabelle Damiani, Nicolas Pauly, Alain Puppo, Renaud Brouquisse, Alexandre Boscari

**Affiliations:** Institut Sophia Agrobiotech, UMR INRA Université Nice Sophia Antipolis CNRSSophia Antipolis, France

**Keywords:** legume, nitric oxide, nitrogen fixation, *Rhizobium*, symbiosis

## Abstract

The symbiotic interaction between legumes and nitrogen-fixing rhizobium bacteria leads to the formation of a new organ, the nodule. Early steps of the interaction are characterized by the production of bacterial Nod factors, the reorientation of root-hair tip growth, the formation of an infection thread (IT) in the root hair, and the induction of cell division in inner cortical cells of the root, leading to a nodule primordium formation. Reactive oxygen species (ROS) and nitric oxide (NO) have been detected in early steps of the interaction. ROS/NO are determinant signals to arbitrate the specificity of this mutualistic association and modifications in their content impair the development of the symbiotic association. The decrease of ROS level prevents root hair curling and ITs formation, and that of NO conducts to delayed nodule formation. In root hairs, NADPH oxidases were shown to produce ROS which could be involved in the hair tip growth process. The use of enzyme inhibitors suggests that nitrate reductase and NO synthase-like enzymes are the main route for NO production during the early steps of the interaction. Transcriptomic analyses point to the involvement of ROS and NO in the success of the infection process, the induction of early nodulin gene expression, and the repression of plant defense, thereby favoring the establishment of the symbiosis. The occurrence of an interplay between ROS and NO was further supported by the finding of both S-sulfenylated and S-nitrosylated proteins during early symbiotic interaction, linking ROS/NO production to a redox-based regulation of the symbiotic process.

## Introduction

Symbiosis describes a situation in which two or more organisms belonging to different species live together for an extended period of time (De Bary, 1879). Both partners can influence the fate of symbiosis, from the host side by the intensity of the immune response, and from the symbiont side by the degree of biological perturbations inflicted on the host (Moné et al., 2014). An important factor for the evolution of symbiosis is the control of redox environment (Hentschel et al., 2000). Redox homeostasis must be tightly controlled to stay under the situation of oxidative stress and to act as signaling pathway. The chemical instability of reactive oxygen species (ROS) and reactive nitrogen species in living organisms is an important property which explains their multi-faceted roles in biology, particularly in plant–microbe interactions (Mittler et al., 2011; Puppo et al., 2013; Hichri et al., 2015; Meilhoc et al., 2015).

The symbiotic associations between Legumes (Fabaceae) and bacteria of *Rhizobium* type implies a recognition step which ultimately leads to the formation of nitrogen-fixing structures called nodules (Oldroyd and Downie, 2008). The interaction starts with the secretion of flavonoids by the plant roots. The perception of the flavonoids by the bacteria leads to the production of bacterial lipochito-oligosaccharides (Nod factors, NF), their specific recognition by the plant, and the induction of nodulation genes in both partners (Oldroyd and Downie, 2008). Increasing evidence support the critical role of ROS and nitric oxide (NO) in the recognition, signaling and immunity processes during the first steps of the symbiotic association between the two partners (Puppo et al., 2013). Both ROS and NO accumulate in roots and growing nodules according to specific spatiotemporal patterns, and regulate the expression of numerous genes that govern the development and the set-up of mature nodules. Several comprehensive reviews highlight the different functions played by these molecules in the nitrogen-fixing symbiosis (NFS) depending on the bacterial or plant origin (Boscari et al., 2013b; Puppo et al., 2013; Hichri et al., 2015; Meilhoc et al., 2015). In the present review, much attention will be paid on recent advance in the occurrence and the function of ROS and NO during the initial steps of NFS, and particularly on the specific role of NADPH oxidases, nitrate reductases (NRs) and hemoglobins (Hbs) in the control of the balance between ROS/NO production and catabolism.

## Role of ROS in the Establishment of the Symbiotic Interaction

### Involvement of NADPH Oxidase

Reactive oxygen species are transiently produced during rhizobial infection (Peleg-Grossman et al., 2009, 2012). The inhibition of ROS production by the NAD(P)H oxidase inhibitor diphenyleneiodonium (DPI; Peleg-Grossman et al., 2007), and the correlation between ROS accumulation and transcript accumulation of two NADPH oxidase genes in response to NF in *Medicago truncatula* roots (Lohar et al., 2007), argue for the involvement of NADPH oxidases in ROS generation. NADPH oxidase genes, also named respiratory burst oxidase homologs (Rbohs), were recently identified and characterized in legume genomes (Lohar et al., 2007; Marino et al., 2011; Montiel et al., 2012). In *Phaseolus vulgaris*, nine *Rboh* genes have been identified (Montiel et al., 2012). *PvRbohB* is particularly accumulated in shoots, roots and nodules. Promoter activity of *PvRbohB* was detected during infection thread (IT) progression and nodule development. Transgenic roots knocked-down for *PvRbohB* by RNA interference (RNAi), Montiel et al. (2012) showed a reduced ROS production with concomitant reduction of nodulation. Microscopy analysis revealed that progression of the ITs was affected in *PvRbohB*-RNAi roots indicating that RbohB could play a key role in successful rhizobial colonization and proper IT growth and shape (Montiel et al., 2012). Ten *Rboh* genes were identified in *M. truncatula* genome (Marino et al., 2012). Their involvement in H_2_O_2_ synthesis during root infection by *Sinorhizobium meliloti* needs to be fully evidenced, but downregulation of legume *Rbohs* leads to decreased nodulation efficiency and an impairment of nitrogen fixation (Marino et al., 2011; Puppo et al., 2013).

The involvement of other potential enzymatic ROS sources cannot be excluded. Type III peroxidases (Prx-III), which were implicated in generation of apoplastic ROS are good candidates (Martinez et al., 1998; Bindschedler et al., 2006). They were reported to promote cell wall hardening (Passardi et al., 2004) and rigidification of IT cell wall and matrix (Wisniewski et al., 2000). Whole transcriptome analyses reveal that Prx-III genes, which were named *rhizobial induced peroxidases* (*Rip1-10*), are inducible by rhizobia and NFs in root hairs (Breakspear et al., 2014), suggesting that they could be involved in root hair ROS production during plant-symbiont recognition.

### ROS in Root Hair Response to *Rhizobium* and NF

A rise of ROS was observed in root cortical cells of inoculated *M. truncatula* plants, which peaked at 24 h after inoculation and remained high after 48 h (Peleg-Grossman et al., 2009, 2012). During the infection process, production of superoxide anion (O_2_^-^) and H_2_O_2_ was localized in IT and infected cells (Santos et al., 2001; Ramu et al., 2002; Rubio et al., 2004). In *P. vulgaris*, a transient increase of ROS was detected at the tip of root hairs within seconds after NF addition (Cardenas et al., 2008). However, after several minutes H_2_O_2_ production appears to be inhibited by NF (Shaw and Long, 2003; Lohar et al., 2007). ROS production was not observed in *M. truncatula* plants inoculated with a *S. meliloti nodD1ABC* mutant unable to produce NF, indicating that the oxidative burst is activated downstream NF perception (Ramu et al., 2002). Furthermore, suppression of immune responses (ROS production, SA accumulation) was observed in *M. truncatula* and *M. sativa* roots upon addition of NF (Martínez-Abarca et al., 1998; Shaw and Long, 2003). It was suggested that ROS production is necessary for infection initiation, but prolonged and elevated levels are detrimental to nodulation (Toth and Stacey, 2015). The subsequent hypothesis is that NF may activate a first ROS production wave involved in nodule development, and inhibit a second one involved in defense reactions. The first wave would modulate the expression of plant genes and/or the redox status of proteins involved in root hair deformation (Lohar et al., 2007), IT progression and nodule formation (Montiel et al., 2012; Puppo et al., 2013).

Moreover, H_2_O_2_ appears to control a key step of the interaction. An *S. meliloti* strain, overexpressing a catalase gene, showed a delayed nodulation phenotype associated with aberrant IT (Jamet et al., 2007). The catalase overexpression probably decreased the internal H_2_O_2_ concentration of the bacteria progressing inside the IT, as observed in free-living conditions. Thus, a positive role for H_2_O_2_ during IT elongation was proposed that could be related to IT rigidity (Rathbun et al., 2002), or to a cytoplasmic signal used by the bacteria to regulate symbiotic function (Pauly et al., 2006). Alternatively, specific posttranslational H_2_O_2_ protein modifications might occur in IT as observed for nitrogen fixing bacteroids (Oger et al., 2012).

## Role of NO in the Establishment of the Symbiotic Interaction

### NO Production

**FIGURE 1:**
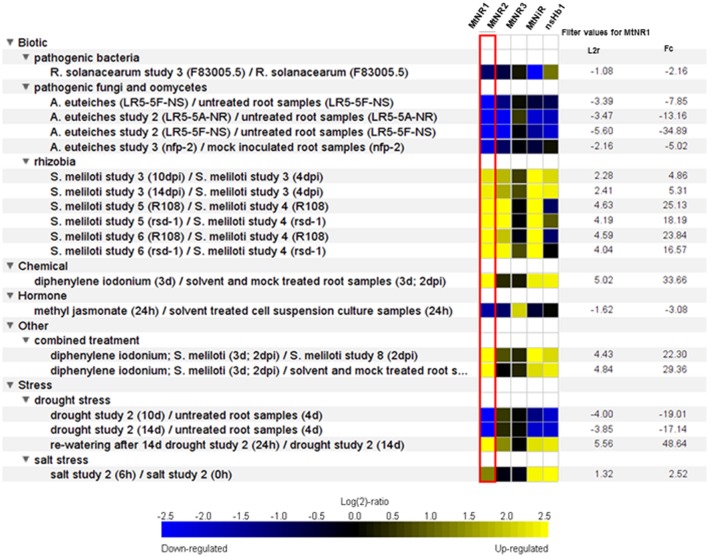
**Screenshot of the results obtained with the Genevestigator Perturbation tool for the *Medicago truncatula* MtNR1 (MTR_3g073170), MtNR2 (MTR_5g059820), MtNR3 (MTR_3g073150), MtNiR (MTR_4g086020), ns-Hb1 (MTR_4g068860) genes on the Affymetrix 61K Medicago GeneChip.** The “Heatmap-List” view is presented here. Perturbations are ordered according to the expression log2-ratios of MtNR1 and are filtered to keep only the perturbations leading to a atleast twofold-change and a *p*-value of maximum 0.05. Perturbations having the highest log2 ratio are listed first. *R. solanacearum*, *Ralstonia solanacearum*; *A. euteiches*, *Aphanomyces euteiches*; *S. meliloti*, *Sinorhizobium meliloti*.

Several possible pathways of NO synthesis have been reported in plants which can be divided into oxidative (NO synthase like –NOS-like, polyamine-mediated, hydroxylamine-mediated) and reductive (NR, plasma membrane-bound nitrite NO reductase, mitochondrial electron transfer chain, xanthine oxidoreductase) pathways (Gupta et al., 2011; Mur et al., 2013). Two studies investigated potential NO source in the first step of NFS. In the first one, the treatment of soybean roots inoculated with *Bradyrhizobium japonicum* with the NOS inhibitor *N*_w_-nitro-*L*-arginine (*L*-NNA) resulted in a 70% reduction of nodule number, suggesting the contribution of NOS-like enzyme in NO production (Leach et al., 2010). The other report shows that treatment of *M. truncatula* inoculated roots with tungstate, a NR inhibitor, mimics the addition of NO scavenger on the transcriptional regulation of genes involved in the nodulation process, whereas treatment with the NOS inhibitor L-NG-nitroarginine methyl ester (L-NAME) is ineffective (Boscari et al., 2013a). *M. truncatula* genome possess 3 NR genes, *MtNR3* being only expressed during the nodulation process (Puppo et al., 2013). *MtNR1* and *MtNR2* are strongly induced during nodulation process (**Figure 1**). These results suggest a specific role of these enzymes, as a NO source, during symbiosis establishment. The potential involvement of the other plant NO sources was not yet investigated.

In symbiotic bacteria, the main route for NO production is the denitrification pathway, which occurs both in free living bacteria under microoxic conditions and in nodules (Meilhoc et al., 2011). The other way for NO production could be NOS-like enzymes which activity was reported in many bacteria (Sudhamsu and Crane, 2009). In *S. meliloti*, a L-arginine-dependent NO production was reported in free-living cells, although no corresponding gene was found in its genome (Pii et al., 2007). To date, there is no evidence for an involvement of the bacterial partner in NO production during symbiosis establishment.

### NO Metabolism

Toxic or signaling effects of NO depend on its concentration at the site of action (Mur et al., 2013). Therefore, NO concentration should be regulated to allow signaling functions to occur and to limit toxic effects. Hbs are known to act as NO scavengers in plants (Gupta et al., 2011). Based on their sequence homology and affinity for O_2_, plant Hbs have been classified into non-symbiotic hemoglobins (ns-Hbs), leghemoglobins, (Lbs), and truncated hemoglobins (tr-Hbs) (Gupta et al., 2011; Hill, 2012). The three Hb types are expressed in legumes (Nagata et al., 2008; Bustos-Sanmamed et al., 2011). Due to their very high affinity for O_2_ and NO (*Km* in the range of 1–20 nM), ns-Hbs and Lbs are able to scavenge O_2_ and NO traces to convert them to nitrate (Hill, 2012). Lbs, which accumulate to millimolar concentration in infected nodule cells (Appleby, 1992), are thought to buffer free O_2_ in the nanomolar range, avoiding inactivation of nitrogenase while maintaining high O_2_ flux for respiration (Ott et al., 2005). Tr-Hbs are induced in *M. truncatula* (Vieweg et al., 2005), and *Frankia* spp. (Niemann and Tisa, 2008; Coats et al., 2009) during NFS. Based on their expression pattern, it was also suggested that they could be involved in NO homeostasis. Other NO metabolizing pathways such as *S*-nitrosoglutathione reductase (GSNOR), glutathione peroxidase and thioredoxin systems, which are known to also regulate NO level in plants (Leterrier et al., 2011; Correa-Aragunde et al., 2015) have been evidenced in legumes (Renard et al., 2011; Matamoros et al., 2015), but their respective contributions to NO balance remains to be investigated in NFS.

Three Hb classes have been also described in bacteria: flavo-Hb (Hmp), single-domain Hb (sd-Hb), and truncated Hb (tr-Hb), which exhibit NO scavenging and detoxification activity (Sanchez et al., 2011). The lower competitiveness of *S. meliloti* overexpressing *hmp* strains as compared to the WT is an argument in favor of a Hmp role in NO control during the infection process (del Giudice et al., 2011). The respiratory NO reductase (Nor) and two proteins of the NnrS family (NnrS1 and NnrS2) were shown to be also involved in NO degradation and to be essential in maintaining efficient NFS (Cam et al., 2012; Meilhoc et al., 2013; Blanquet et al., 2015). However, their role in NO control during symbiosis establishment was not yet investigated.

### NO in the Recognition of *Rhizobium* Partner

Numerous findings support the hypothesis that NO signaling plays a role in plant microbe recognition. Treatments of soybean roots with L-NNA reduced nodule number during interaction with *B. japonicum*, a phenotype reverted by the addition of the NO-donor diethylenetriamine NONOate (Leach et al., 2010). In the same way, the decrease of the NO content by 2-[4-carboxyphenyl]-4,4,5,5-tetramethyl imidazoline-1-oxyl-3-oxide (cPTIO) treatment and by *hmp* overexpression in the plant partner delayed nodulation in the *M. truncatula – S. meliloti* interaction, indicating that NO is required for an optimal establishment of the symbiotic process (del Giudice et al., 2011). Several reports underlined the role of plant Hbs in regulating NO level during symbiosis establishment. First, upon *Lotus japonicus* inoculation with *Mesorhizobium loti*, a transient production of NO was observed at the root surface 4 h post-inoculation (hpi), which then decreased to its basal level 10 hpi (Nagata et al., 2008). However, when *L. japonicus* was infected with the plant pathogens *Ralstonia solanacearum* and *Pseudomonas syringae*, NO was continuously produced for at least 24 hpi (Nagata et al., 2008). The decrease in NO level after its transient accumulation following infection with *M. loti* was assigned to *LjHB1* which gene expression was upregulated by the symbiont, but not by the pathogens (Nagata et al., 2008, 2009; Murakami et al., 2011). In addition, NO was shown to up-regulate ns-Hb expression in different plant species (Shimoda et al., 2005; Sasakura et al., 2006; Nagata et al., 2008). These observations suggest that at early step of symbiotic interaction, the initial burst of NO induces the expression of ns-Hb that, in return, scavenges NO and down-regulates its level to lower plant defense response and allow the reception of the symbiont. Experimental evidence showed that lipopolysaccharides from *M. loti* induced *LjHB1* expression and NO production in *L. japonicus* roots (Nagata et al., 2009; Murakami et al., 2011). They showed the polymer distributed on the outer membrane of Gram-negative bacteria play a major role in the recognition and establishment of symbiosis.

Nitric oxide production was also observed in the infection pockets, along the ITs, and in dividing cortical cells of the nodule primordia (del Giudice et al., 2011). The presence of NO in nodule primordia present high similarity with the local NO increase observed in lateral root primordia (Correa-Aragunde et al., 2004; Lanteri et al., 2006). In both studies, authors reported that NO could modulate the expression of cell cycle regulatory genes (Correa-Aragunde et al., 2006; del Giudice et al., 2011). It was notably observed in *M. truncatula* that NO scavenging provokes the down regulation of plant genes involved in nodule development, such as *MtCRE1* and *MtCCS52A* (del Giudice et al., 2011). Furthermore, transcriptomic analysis of cPTIO-treated inoculated roots of *M. truncatula* showed that NO is involved in the regulation of many family of genes related to cell cycle process and protein synthesis in nodule primordia (Boscari et al., 2013a), which reinforces the hypothesis that NO plays a similar function in nodule and lateral root organogenesis.

**FIGURE 2:**
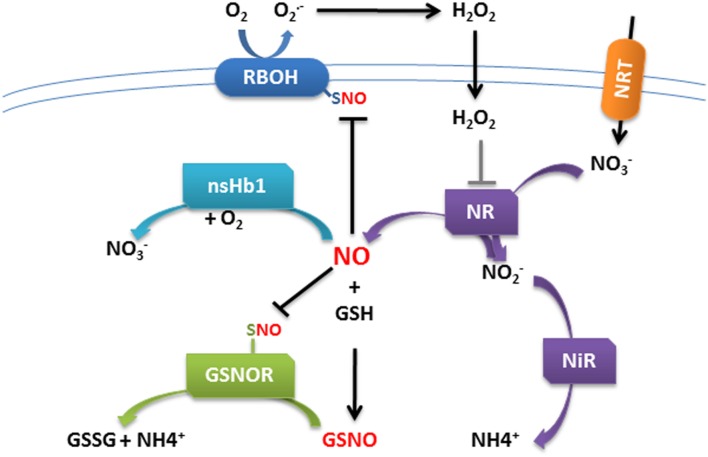
**Schematic model of cross-talk regulation between ROS and NO in plant cells.** H_2_O_2_ produced by NADPH oxidase leads to the activation of nitrate reductase (NR) and concomitant NO production. NO accumulation in turn blunts NADPH oxidase activity by *S*-nitrosylation, preventing accumulation of excess ROS. In the nitrogen assimilation pathway, nitrate (NO_3_^-^) is taken up by nitrate transporters (NRT) and reduced to nitrite (NO_2_^-^) by NR. Nitrite is reduced to NO by NR. NO reacts with reduced glutathione (GSH) in the presence of O_2_ to form *S*-nitrosoglutathione (GSNO). GSNO is converted by the enzyme GSNO reductase (GSNOR) into oxidized glutathione (GSSG) and NH_3_. NO accumulation leads to GSNOR *S*-nitrosylation and inhibition preventing GSNO degradation.

Interestingly, the control of NO homeostasis through the spatiotemporal coordination of NR and Hb gene expression was recently hypothesized to participate to nitrate sensing in maize roots (Trevisan et al., 2015). Moreover, Frungillo et al. (2015) demonstrate that NO is at the center of nitrogen homeostasis in *Arabidopsis* plants. They demonstrated that NO derived from nitrate assimilation inhibits the activity of GSNOR, which controls the cellular levels of GSNO, by *S*-nitrosylation (addition of a NO group to cysteine thiols) of some of its cysteine residues. They observed that inhibition of GSNOR is necessary to amplify a SNO signal, which in turn feedback regulates nitrate assimilation (**Figure 2**). It is noteworthy that nodulation efficiency is as well finely tuned according to the nitrate availability (Streeter, 1988).

## NO and ROS Interplay

Overall, it appears that ROS and NO present a fine-tuned spatio-temporal modulation which plays a critical role in signaling and immunity in the associations between Legumes and rhizobia. The occurrence of an interplay between ROS and NO was supported by the finding of both *S*-sulfenylated and *S*-nitrosylated proteins posttranslational modification during NFS (Oger et al., 2012; Puppo et al., 2013), linking ROS/NO production to a redox-based regulation of the symbiotic process. Peroxynitrite (ONOO^-^) is a signaling molecule formed when NO reacts with O_2_^-^. Its function may be mediated by the selective nitration of Tyr residues in a small number of proteins. Blanquet et al. (2015) reported that glutamine synthetase GS1a, a key enzyme in N_2_-fixation, is nitrated in nodules. Such NO/ONOO^-^-mediated posttranslational inactivation of GS provides a direct link between NO/O_2_^-^ signaling and nitrogen metabolism in root nodules. Whether these molecules work in synergy in early symbiosis steps is not determined yet, however, the comparison of *M. truncatula* inoculated roots treated with either the NADPH oxidase inhibitor (Andrio et al., 2012), or the NO scavenger (Boscari et al., 2013a), reveals a strong overlap in the signaling pathways triggered by either molecule. Furthermore 75% of the 316 differentially regulated genes identified in both analyses are similarly up- or down-regulated (Puppo et al., 2013). Amongst the up-regulated genes, some gene families involved in plant defense and secondary metabolism were notably identified, as previously highlighted during cell death induction (Zago et al., 2006). Moreover, in transcriptomic analysis of DPI-treated *M. truncatula* roots (Andrio et al., 2012), *MtNR1*, *MtNiR*, and *Mtns-Hb1* were found strongly up-regulated (**Figure 1**), suggesting that H_2_O_2_ could control the transcriptional regulation of enzymes involved in NO homeostasis. In the same way, it was recently reported that an elevated ROS concentration during plant–pathogen interaction results in the activation of NR (Wang et al., 2010; Lin et al., 2012). Interestingly, in *Arabidopsis* leaves, NR activation leads to increased NR-mediated NO production, and to the subsequent inactivation of NADPH oxidase activity by *S*-nitrosylation during pathogen infection (Yun et al., 2011). These results evidence the overlapping connection between NO and ROS production with a negative feedback loop of the NO on ROS production (**Figure 2**). However, such type of ROS/NO interplay still has to be clearly elucidated in roots and during establishment of NFS.

## Conclusions and Future Directions

We showed that spatio-temporal accumulations of ROS and NO are critical for the specificity of their function throughout the successive steps of symbiosis initiation. NADPH oxidases were identified as major source of ROS production, and NR and NOS-like have been evidenced as NO sources during the early steps of the interaction. Importance of plant and bacterial Hbs in NO balance was particularly highlighted. However, the involvement of alternative systems like catalase, glutathione peroxidase, GSNOR and thioredoxins remains to be investigated to decipher their respective contribution in ROS and NO balance. Transcriptomic analyses point to the involvement of ROS and NO in the success of the infection process notably by the repression of plant defense responses favoring the establishment of the symbiosis Further investigations will aim to decipher the possible regulation of nodule NADPH oxidase activity by NO and the transcriptional regulation of genes involved in NO homeostasis by H_2_O_2_ in symbiosis establishment.

## Author Contributions

ID, NP, and AP contribute to the writing of the different paragraph about ROS. RB and AB contribute to the writing of the different paragraph about NO and interplay ROS/NO.

## Conflict of Interest Statement

The authors declare that the research was conducted in the absence of any commercial or financial relationships that could be construed as a potential conflict of interest.
